# Lipid rafts in T cell signalling and disease

**DOI:** 10.1016/j.semcdb.2007.08.002

**Published:** 2007-10

**Authors:** Elizabeth C. Jury, Fabian Flores-Borja, Panagiotis S. Kabouridis

**Affiliations:** a Centre for Rheumatology, Royal Free and University College Medical School, University College London, London W1P 4JF, United Kingdom; b Bone and Joint Research Unit, Queen Mary's School of Medicine and Dentistry, Charterhouse Square, London EC1M 6BQ, United Kingdom

**Keywords:** Lipid rafts, T cell, Lupus, Autoimmunity, Review

## Abstract

Lipid rafts is a blanket term used to describe distinct areas in the plasma membrane rich in certain lipids and proteins and which are thought to perform diverse functions. A large number of studies report on lipid rafts having a key role in receptor signalling and activation of lymphocytes. In T cells, lipid raft involvement was demonstrated in the early steps during T cell receptor (TCR) stimulation. Interestingly, recent evidence has shown that signalling in these domains differs in T cells isolated from patients with autoimmune diseases such as systemic lupus erythematosus (SLE) and rheumatoid arthritis (RA). Here, we discuss these findings and explore the potential of lipid rafts as targets for the development of a new class of agents to downmodulate immune responses and for the treatment of autoimmune diseases.

## 1 Signalling by the T cell receptor (TCR)

The TCR recognizes peptides presented in the context of appropriate major histocompatibility complex (MHC) proteins expressed on the surface of antigen presenting cells (APCs). Signalling cascades that branch out of the stimulated TCR in conjunction with signals that originate from concomitantly stimulated co-receptors result in the activation of the T cell and development of effector functions [1].

The immediate outcome of TCR stimulation is tyrosine phosphorylation of immunoreceptor tyrosine-based activation motifs (ITAMs) present in the cytoplasmic domains of the chains (γ, δ, ɛ, and ζ) that comprise the TCR-associated CD3 complex [2,3]. ITAM phosphorylation is mediated by the Src-family tyrosine kinase Lck [4,5]. The phosphorylated TCR recruits to the plasma membrane the cytosolic ZAP-70 tyrosine kinase [6], the main substrate of which is the transmembrane adaptor LAT (linker for activation of T cells). In turn, LAT phosphorylation triggers the formation of multi-protein signalling complexes at the plasma membrane. Proteins that participate in such assemblies include other scaffold molecules such as Grb-2, SLP-76, and Gads, and enzymes like phospholipase C (PLC) γ1 and phosphoinositide 3-kinase (PI3K) [7–9]. The LAT-nucleated complexes at the cytoplasmic face of the plasma membrane control downstream signalling pathways that lead to changes in cell morphology and gene transcription.

Recognition of antigen/MHC complexes by the TCR is assisted by co-receptors, CD4 and CD8, and co-stimulatory receptors such as CD28, expressed on the surface of T cells that pair with their cognate ligands on the APC. The coordinated action of these interactions and ensuing signalling result in a stable, yet dynamic, zone of contact between the two cells which has been named the immunological synapse (IS) (reviewed in Ref. [10]). The plasticity of the IS became apparent through imaging studies showing temporal and spatial recruitment of various co-receptors and signalling molecules within subdomains of the synapse [11]. For example, in the mature IS, the central region of the synapse, where the T cell and APC membranes are in close proximity, concentrates the TCR, the CD4 and CD28 receptors, and associated signalling molecules such as Lck and protein kinase C (PKC) θ [12]. Larger and heavily glycosylated molecules such as CD44, CD45 and CD43 are excluded from the central region and preferentially occupy a peripheral area that surrounds the core of the synapse [13,14]. Lipid rafts are found to accumulate at the IS, as was reported by staining the cells with cholera toxin B subunit (CTB), which binds to Ganglioside M1 (GM1), a marker of raft membranes [15,16]. Furthermore, using the fluorescent probe Laurdan, condensation of the plasma membrane was detected at the site of the IS, suggesting accumulation of lipid rafts [17]. Interestingly, the tyrosine phosphatase CD45 has been shown to transiently move to the proximity of the TCR at the later stages during T cell/APC interaction [18]. However, initial TCR activation and early tyrosine phosphorylation precedes formation of the IS and occurs in TCR-containing microclusters which form within seconds of the initial TCR-MHC/antigen contact and contain activated Lck, ZAP-70, and LAT [19,20]. Subsequent formation of the IS not only provides the sustained signal needed for gene transcription but may also control the eventual cessation of the signal and downregulation of the immune response.

## 2 Lipid rafts in T cells; size matters

**Fig. 1:**
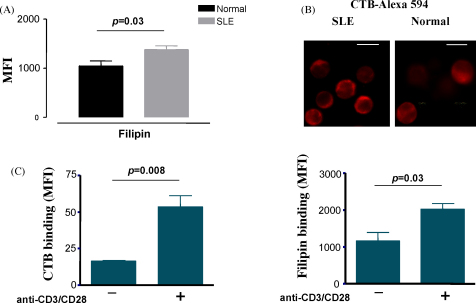
Increased levels of cholesterol and GM1 in the plasma membrane of T cells from patients with SLE and activated normal T cells. (A) Purified T cells from SLE patients and healthy controls were labelled with the cholesterol chelator filipin and analysed by flow cytometry. The results shown are the cumulative data collected from five lupus patients and five healthy volunteers. (B) SLE and normal T cells were stained with Alexa-fluor 594-conjugated cholera toxin B subunit (CTB-Alexa 594) and analysed by confocal microscopy. The images shown are adjusted to the same output intensity and are representative of samples generated from two healthy donors and two SLE patients. Bar = 10 μm. (C) Peripheral blood T cells from healthy donors were activated *in vitro* with a combination of anti-CD3/anti-CD28 antibodies and stained with CTB-Alexa 594 or filipin to assess the levels of GM1 and cholesterol, respectively.

The term lipid raft is used to describe microdomains in the plasma membrane that are in liquid-ordered phase owing to their lipid composition which is rich in cholesterol, glycosphingolipids, and sphingomyelin [21–23]. As a result, their structural properties are different from the glycerophospholipid-rich bilayer which comprises the bulk of the plasma membrane [24]. Although it is agreed that in unstimulated cell lipid rafts are too small to resolve with the light microscope, their actual size and protein composition is an area of ongoing debate [25,26]. While some groups report a size between 50 and 200 nm [27], others have measured a much smaller size of <20 nm [28]. This is a key issue that needs to be addressed if we are to better understand their function (the reader is also referred to the accompanying reviews in this issue). For example, microdomains of a larger size will promote compartmentalization of plasma membrane proteins that have higher affinity for the liquid-ordered phase, while they will exclude proteins that have higher affinity for the liquid-disordered phase. In this scenario, some protein–protein interactions will be favoured while others will be prevented. This arrangement may change upon physiological stimulation of receptors such as the TCR. This model has been the ‘classical’ raft hypothesis. On the other hand, if lipid rafts are of smaller size (<20 nm) they will be able to accommodate only few protein molecules. Although it is difficult to see how such ‘nanodomains’ could induce any significant segmentation of the membrane, a recent computational modeling study on raft–protein dynamics indicated that small, mobile rafts can reduce the diffusion rate of associated proteins and hence increase protein–protein collisions. In contrast, proteins that are excluded from rafts did not show reduced mobility [29]. Furthermore, coalesence of these small, highly mobile, and probably short-lived, units into larger structures under certain conditions, such as receptor triggering, will enable them to adopt the properties of larger rafts as mentioned above [30]. Larger rafts are likely to be controlled by endocytosis unless stabilized by, for example, receptor stimulation [31]. Receptor-induced protein–protein interactions also have a critical role and will further facilitate aggregation and signalling [32]. In this model, any change in the status of the lymphocyte that results in higher accumulation of lipids which are constituents of raft membrane, is likely to profoundly change membrane organization by supporting formation of larger rafts. These changes may in turn influence the strength or duration of receptor signal. Thus, inappropriate changes in the size and/or structure of lipid rafts could influence their stability and may result in abnormal signalling and pathological conditions. Changes in the level of membrane cholesterol and GM1 have been reported in activated and autoimmune lymphocytes (see Fig. 1 and Section 4 below).

## 3 Lipid rafts in TCR signalling

Historically, the interest of immunologists in T cell lipid rafts originated from the observation that Lck, which phosphorylates the TCR ITAMs, localizes to these domains owing to its N-terminal dual acylation [33–35]. It was shown that the ability of the kinase to support TCR signalling critically depends on its lipid modification [35,36]. Soon after, LAT was found to partition to raft domains through palmitoylation of two cysteine residues that are in juxtaposition to its transmembrane segment [7]. As with Lck, mutation of the cysteines delocalizes LAT and negates its biological function [37].

Like all the Src-family kinases, Lck activity is regulated by the phosphorylation-dephosphorylation cycle of two tyrosine (Y) residues located in the catalytic domain (Y394) and the C-terminus (Y505) of the protein [38]. Phosphorylation of Y505 by Csk (C-terminal Src kinase) [39] induces its interaction with the Src homology (SH) 2 domain of the protein causing the folding of the molecule into a ‘closed’ conformation which has low enzymatic activity [40,41]. In contrast, autophosphorylation of Y394 forces the unfolding of the protein into an ‘open’ configuration which has substantially higher activity [42].

The activity of Lck is also regulated by CD45, a receptor-type tyrosine phosphatase which dephosphorylates Y505 [40,43]. The constitutive action of CD45 maintains a pool of Lck in a primed form ready to support signalling by the TCR. The importance of CD45 has been highlighted by the absence of TCR signalling in mice and cell lines that are deficient in its expression [44,45]. It is important, however, to note that CD45 also has a negative role in signalling by dephosphorylating Lck at Y394 [46] and the CD3ζ chain [47]. Initially it was suggested that CD45 is excluded from raft domains [48]; however, subsequent reports have identified a small but detectable fraction of the phosphatase in these domains [49,50]. How the cell balances the positive and negative actions of CD45 during the early steps of TCR signalling is not yet clear. A current theory that explains its dual role suggests that, while the phosphatase constitutively maintains a pool of Lck primed for action, it is excluded from the area of TCR engagement upon stimulation of the receptor, due to its large, heavily glycosylated extracellular domain, which cannot be accommodated into the narrow space created by the close proximity of the T cell and APC surfaces [13,51]. Accumulation of lipid rafts and of Lck in these areas will shift the balance towards increased phosphorylation and signal transduction [17,52]. This theory is supported by experiments showing that CD45 chimeras in which the extracellular portion has been substituted by domains of progressively smaller size, gradually lose their ability to support TCR signalling [50]. Furthermore, it is known for many years that the various isoforms of CD45 which differ in their extracellular domain also differ in their ability to support TCR signalling [43].

**Fig. 2:**
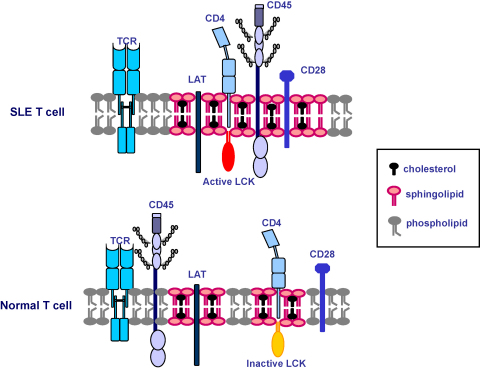
Differential association of signalling molecules to lipid raft domains in SLE may contribute to a breakdown in peripheral T cell tolerance. Higher synthesis of cholesterol and of other lipids could result in larger or more stable lipid raft domains. These changes in the plasma membrane could be the reason for the increased co-localization of CD45 phosphatase with lipid rafts and the higher Lck activity seen in SLE T cells. Enhanced protein–protein interactions and changes in enzymatic activity could lower the threshold for TCR activation.

A finding which at first glance seems to contradict this theory is the observation that the pool of Lck present in lipid rafts is phosphorylated on Y505 and is primarily in its ‘closed’ conformation [48,53–55]. This could be due to the presence of the PAG-Csk inhibitory complex in lipid rafts. PAG (protein associated with GEMs or otherwise known as Csk binding protein (Cbp)) is a transmembrane adaptor which, like LAT, is palmitoylated on two membrane-proximal cysteines [56,57]. This adaptor is phosphorylated by Src-family kinases and recruits Csk to lipid rafts [55,58,59]. Recruited Csk in turn phosphorylates Lck at Y505 inducing its ‘closed’ conformation [55,59]. Recently, however, the generation of PAG-deficient animals has revealed that recruitment of Csk to the membrane fraction and Lck Y505 phosphorylation is not significantly reduced in T cells, suggesting that an additional, yet unidentified, Csk anchor exists [60]. Under certain conditions, such as receptor stimulation, a tyrosine phosphatase, possibly CD45, may transiently associate with lipid rafts dephosphorylating Y505 and converting raft-associated Lck molecules into the ‘open’ structure (Fig. 2, top panel) [61]. Interestingly, chimeras of CD45 which show better localization to lipid rafts are able to support TCR signalling more efficiently [50]. Lipid rafts containing primed Lck subsequently associate with the microclusters of engaged TCRs, phosphorylating ITAMs and initiating signal transmission [62]. In this scenario formation of TCR microclusters should precede phosphorylation of the ITAMs by Lck. In support of this model Campi et al. found that, in cells treated with inhibitors of Src kinases, microclusters of engaged TCRs were able to form, although tyrosine phosphorylation was abolished [19]. Rather, microcluster formation was dependent on actin polymerization [19]. Significantly, lipid raft microdomains were found to contain actin and other cytoskeletal proteins suggesting that their movement may be regulated by reorganization of the actin cytoskeleton [63–66]. Subsequent formation of the IS will promote a wider segregation of receptors and signalling proteins excluding CD45 from the area of TCR phosphorylation allowing for sustained signalling. Therefore, compartmentalization of CD45 both inside and outside of lipid rafts could be a key issue in determining not only the threshold but also the duration of TCR activation during an immune response.

## 4 Lipid raft signalling in autoimmune T cells

The homeostasis of the immune system is stringently controlled by the specificity and fidelity of lymphocyte activation. In autoimmune diseases this specificity and fidelity is compromised leading to pathology. Whether changes in the composition or structure of lipid rafts play a role in autoimmunity is an important question which has started to be addressed in the last few years. It was noted that human T cells activated *in vitro* via their TCR synthesize more GM1 lipid, a component of raft domains, as detected by staining with CTB [67,68] and Fig. 1C). The physiological significance of this observation is not yet clear; however, it is possible that higher GM1 and cholesterol content report on an increased portion of the plasma membrane being in the liquid-ordered phase. As a result the lateral mobility of receptors and other signalling molecules may be reduced, increasing compartmentalization of proteins at the plasma membrane. Such changes may impact on thresholds for activation of receptors and possibly of the TCR. Indeed, over-loading healthy T cells with cholesterol reduces membrane fluidity and disrupts the interaction of signalling molecules [69]. Interestingly, plasma membrane cholesterol has been linked to increased T cell immunosenescence associated with aging [70]. It is not known whether the mobility of molecules associated with glycosphingolipids such as glycosylphosphatidylinositol (GPI)-linked receptors, is altered in resting and activated cells.

Similarly, the plasma membrane of peripheral blood T cells freshly purified from patients suffering from the autoimmune disease SLE was found to contain more cholesterol and GM1, confirming that these cells have an activated phenotype ([71,72] and Fig. 1). When purified SLE T cells were cultured *in vitro* the levels of cholesterol and GM1 gradually decreased to that seen in naïve T cells from healthy volunteers, indicating that stimuli in the body of lupus patients activate T cells to increase synthesis of these lipids. Higher synthesis of the lipids was reinstated following TCR stimulation. Culturing the cells in the presence of serum from SLE patients did not maintain the high expression of GM1 and cholesterol, suggesting that cell to cell contacts are needed for the activation of SLE T cells in the body [71]. Relevant is the finding that dendritic cells (DCs) from patients have an activated phenotype due to the sustained action of interferon alpha (INFα) [73]. Activated DCs may be instrumental in the presentation of autoantigens contributing to the activation of T cells.

There are documented defects in the expression and function of various signalling molecules and pathways proximal to the TCR in SLE T cells [74,75]. Among these are reduced expression of the canonical ζ chain of the TCR but expression of non-conventional ζ transcripts [76], expression of the γ chain of the Fcɛ receptor [77] and reduced expression of Lck [78]. Reduction in Lck expression was more pronounced in T cells from patients with active disease and was independent of the treatment regime. Reduction of Lck expression is probably due to a combination of reduced gene transcription and increased ubiquitination/degradation of the protein [78,79]. In accordance, SLE T cells when stimulated via the TCR are poor activators of MAPK (mitogen-activated protein kinase) pathways and hypoproliferate *in vitro* [80,81]. Increased consumption of Lck via ubiquitination and degradation may be, at least in part, due to the sustained stimulation of lupus T cells by autoantigens, since it has been shown that sustained TCR stimulation leads to reduction in the expression of Lck [15].

Compared to healthy controls, lipid rafts from SLE T cells were found to contain higher amounts of CD45 and co-immunoprecipitation experiments revealed that a larger fraction of this pool was associated with Lck. As a consequence more Lck in lipid rafts was in the active form [71]. Therefore, an increase in the localization of CD45 into lipid rafts is a key step for Lck conversion to the active form and may be a critical step in the activation of T cells. A similar observation was made with B lymphocytes isolated from SLE patients. Here again, higher expression of GM1 was noted as assessed by CTB binding, and this was accompanied by reduced expression of Lyn, an Src-family kinase responsible for the phosphorylation of the ITAMs in the B cell receptor (BCR) chains [82]. As in T cells, there was an increase in the level of CD45 present in lipid rafts in SLE B cells. Upon activation, CD45 recruitment to the site of activated BCRs was more prolonged in SLE B cells compared to cells from healthy volunteers, as was recruitment of Lyn [83]. Like in T cells, these observations may indicate that B cells in SLE patients are responding to a chronic stimulation by autoantigens.

One way of addressing the importance of higher cholesterol synthesis in TCR signalling is to treat cells with statins. These are small molecule inhibitors of 3-hydroxy-3-methylglutaryl coenzyme A (HMG-CoA) reductase, the rate-limiting enzyme in the biosynthetic pathway of cholesterol. Treatment of SLE T cells with artorvastatin inhibited the co-localization of CD45 and Lck in lipid rafts resulting in the reduction of active Lck [84]. This correlated with restoration of Lck expression back to normal levels and ERK activation following TCR triggering [84]. Collectively these observations suggest that increased synthesis of cholesterol is associated with protein–protein interactions and signalling events that determine the activation status of the lymphocyte. Therefore, pharmacological modification of the structure and/or composition of lipid raft domains may represent a new method to downregulate immune responses in pathological conditions. Statins are already under investigation as potential immuno-modulatory drugs in a range of inflammatory and autoimmune diseases.

A second chronic inflammatory autoimmune disease where T cells may play a role in the pathogenesis is rheumatoid arthritis (RA). In this case, T cells that are purified from the synovial fluid (SF) of inflamed joints, in contrast to T cells from the periphery, were shown to have an activated phenotype, yet they were hyporesponsive to TCR stimuli and proliferated poorly [85,86]. This hyporesponsiveness may be the result of chronic oxidative stress characteristic of the microenvironment of inflamed joints. In T cells, oxidative stress was shown to result in the displacement of LAT from the membrane [87]. This was due to a reduction in the levels of the anti-oxidant glutathione (GSH). Addition of *N*-acetyl-l-cysteine restored LAT localization to the membrane and TCR signalling [87,88]. Furthermore, reactive oxygen species (ROS)-mediated modification of Lck structure has been reported in SF, but not peripheral blood T cells, from RA patients [89]. Therefore, under certain conditions ROS-mediated modification of cysteine residues, which are targets of *S*-acylation, could delocalize proteins from lipid rafts and result in inhibition of activation. This is highlighted by reports showing that mutant mice with reduced capacity to produce ROS due to a polymorphism in the neutrophil cytosolic factor 1 (Ncf1) are more prone to severe arthritis [90]. Furthermore, in T cells, ROS production regulates surface redox levels to suppress autoreactivity and development of arthritis [91]. A similar situation has been observed with tumour infiltrating T cells (TILs) which, like SF T cells, are in a high oxidative stress microenvironment and are defective in their ability to fully mobilize signalling pathways following TCR stimulation.

Taken together, these observations underline the importance of lipid raft localization of proteins such as Lck, LAT and CD45, as central to TCR signalling. Increased synthesis of cholesterol and of other lipids may alter the structure of the plasma membrane of lymphocytes in a way that favours protein interactions and signalling. On the other hand, under certain conditions modification of critical residues in raft-associated proteins will disrupt their localization and inhibit cell activation. In both cases these changes profoundly impact on the function of lymphocytes by either supporting autoimmunity or failing to mount a response, respectively.

## 5 Conclusions and perspectives

A supposition of the lipid raft theory is that cholesterol and certain other lipids play an elementary role in the formation of membrane domains. Results from initial studies investigating *in vitro* activated lymphocytes or lymphocytes isolated from patients with autoimmune diseases show that the levels of cholesterol and GM1 (and possibly of other lipids) are increased compared to healthy cells. It is feasible that changes in lipid composition modify membrane organization possibly by increasing the abundance or the size of lipid raft domains. Alteration of the plasma membrane may be linked to the changes in localization and function of signalling proteins seen in autoimmune lymphocytes, although modulation of other cellular functions such as trafficking, internalization, membrane fusion and others will almost certainly occur, which in turn will have further impact on signalling. This in itself may not be sufficient to start an autoimmune response; however, in conjunction with other predisposing factors it could facilitate initiation of autoimmunity. If this is a correct assumption then it raises the possibility that lipid rafts may be targets for the development of pharmaceuticals to control aberrant immune responses. Statins are obvious candidates for such intervention and their efficacy in autoimmune diseases is currently under investigation [92,93]. On the other hand, sterol-chelating agents, such as the antibiotic nystatin which is used as an anti-fungal treatment, may have the opposite effect by assisting in the activation of lymphocytes [94,95], although detailed studies on its mechanism of action on lymphocytes are limited. Nevertheless, the prospect of lipid raft research moving closer to clinical application is an exciting new development in the field, although remaining controversies and potential new hurdles should not be underestimated.
